# Effectiveness of online learning on health researcher capacity to appropriately integrate sex, gender, or both in grant proposals

**DOI:** 10.1186/s13293-018-0197-3

**Published:** 2018-08-29

**Authors:** Cara Tannenbaum, Krystle van Hoof

**Affiliations:** 1 Institute of Gender and Health, Canadian Institutes of Health Research, 4545 Queen Mary Road, Montreal, Quebec H3W 1W5 Canada; 2 Faculties of Medicine and Pharmacy, Université de Montréal, Montreal, Canada

## Abstract

**Background:**

To describe the effectiveness of online learning to augment academic capacity to consider sex and gender in the conduct of basic science, clinical research, and population health studies.

**Method:**

The analysis compares pre- and post-test scores from 1441 individuals who completed the Canadian Institutes of Health Research Institute of Gender and Health’s interactive e-learning modules between February 2016 and May 2017. The tests measured knowledge, self-efficacy, and self-reported intent to change behavior for three competencies: (1) the ability to appropriately define and distinguish between sex-related versus gender-related variables, (2) the application of methods for integrating sex and gender, and (3) the critical appraisal of sex and gender integration in the design, methods, and analysis plan of research proposals and publications.

**Results:**

Of the 543 individuals who completed the basic science module, 62% demonstrated improved knowledge, and 86% increased self-efficacy across all competencies. Gains in knowledge and self-efficacy also occurred among 84% and 77% of completers of the human data collection module (*n* = 463) and among 73% and 82% of those who completed the secondary data analysis module (*n* = 435). In aggregate, 95% of participants reported an intent to change their behavior with respect to sex and gender in health research.

**Conclusions:**

Interactive online learning combined with feedback and self-assessment results in improved knowledge and self-efficacy for integrating sex and gender in health research.

## Background

New funding agency policies and journal instructions for authors require integration of sex as a biological variable and gender-related social factors in health research [1–4]. The highest standards of excellence in science—including rigor, reproducibility, transparency, and inclusion—are driving this change in order to ensure that all patients benefit from research investments in an equitable fashion. Cell biologists are being asked to record the sex of their cells [5]. Animal experiments are expected to factor sex as a biological variable in the design and methods of the study [6]. Clinical trialists must document the sex and gender of study participants and disaggregate the data in their analyses [3]. Health systems research calls for gender—the socially constructed roles, behaviors, activities, and attributes that a given society considers appropriate for males, females, and other genders—to be a core component of interventions, programs, and policies [7]. Even implementation research acknowledges the advantage of addressing sex and gender to increase the uptake of healthcare solutions [8]. Ultimately, the vision is for evidence-informed sex- and gender-specific recommendations to be integrated into research and clinical practice in a way that best tailors care to the individual patient according to sex, gender, and other identity-related characteristics [9, 10].

The most effective way to educate academics on how to appropriately consider sex and gender as a standard practice in health research remains unknown. Data indicate that uptake has been slow and irregular [11–13]. Barriers include problems with inconsistent terminology, difficulties in applying the concepts of sex and gender, failure to recognize the impact of sex and gender, and uncertainty about how to operationalize these concepts in data collection, analysis, and practice [13]. Attempts to improve research integrity in other areas of science, such as preventing misconduct and reducing publication bias, suggest that overcoming inertia and changing cultural norms are major obstacles [14–16].

Literature on behavior modification among academics points to the success of multimodal education and training combined with interactive audit and feedback [17–19]. The benefits of e-learning are well documented in terms of increased accessibility to education, efficacy, cost-effectiveness, learner flexibility, and interactivity [20]. The assessment of sex and gender in research protocols has never been tested through e-learning and holds promise as a vector for standardizing and building capacity among health researchers across geographic location and career stage.

In 2015, the Institute of Gender and Health of the Canadian Institutes of Health Research developed a series of Internet-based interactive e-learning modules to improve knowledge, skills, and attitude gaps related to sex and gender considerations in health research (http://www.cihr-irsc-igh-isfh.ca). These free, 45-min pedagogical courses were intentionally designed to help researchers and peer reviewers recognize sex- and gender-related mechanisms in health, identify methods for integrating sex and gender variables in health research settings, and critically appraise research protocols and publications based on the integration or omission of sex and gender. Three separate courses were developed: (1) for biomedical research involving animal, cells, or tissues; (2) for primary data collection in research involving human participants; and (3) for the analysis of data from human participants. This report describes the effectiveness of the three sex and gender e-learning modules in supporting improved knowledge, self-efficacy, and behavioral intent among trainees, researchers, peer reviewers, and government employees who completed the courses.

## Methods

### Study design

A quasi-experimental study design was used, comparing pre- and post-questionnaire data from the same participants, prior to and after completion of the online courses.

### Participants

Participants were primarily health researchers, peer reviewers, trainees, research support staff, and government employees. Participants from Canada, the USA, Europe, and Asia completed the modules, either in French or in English. The data presented in this report is from individuals who completed at least one of the three online courses and submitted full data for the pre- and post-evaluations in English or French.

Recruitment of participants occurred through three different channels. The first was through email, newsletters, and social media to researchers already interested in sex and gender science, via a subscription to the Institute of Gender and Health’s communication list. The second method of recruitment was to other researchers through different organizations’ email lists, through presentations and flyers at national and international health research conferences, and via presentations to the chairs of all peer review committees for the Canadian Institutes of Health Research. The third method of enrolling participants included a mandatory requirement for grant applicants to complete one of the training modules in order to be eligible for specific funding opportunities supported by the Institute of Gender and Health. Examples of funding opportunities were catalyst grants to stimulate inclusion of sex as a biological variable for researchers who were previously not accounting for sex or team grants for personalized health research. To support the appropriate integration of sex and gender into their proposals, nominated principal applicants were required to submit a certificate of completion for one of the learning modules to be eligible for funding. Participants were not asked to indicate why they decided to complete the training module, so each individual’s motivation for engaging with the e-learning modules remains unknown.

Completion of one of the online learning modules was required in order to access the post-test. Only data from users who fully completed one of the modules and only responses from their pre-test and post-test questions are included in this analysis.

### Development of the e-learning modules

The content and competencies targeted by each module were informed by consultation with members of the Advisory Board of the Institute of Gender and Health as well as external stakeholders and experts.

Each module was designed to promote three key competencies. The first was the ability to define and distinguish between sex-related and gender-related variables in a health research context. The second was the capacity to recognize when and why sex and gender are relevant considerations in health research and to identify and apply methods for integrating sex and gender in research. The third was to critically appraise research grant proposals and publications on the basis of the integration (or omission) of sex and gender considerations.

**Fig. 1:**
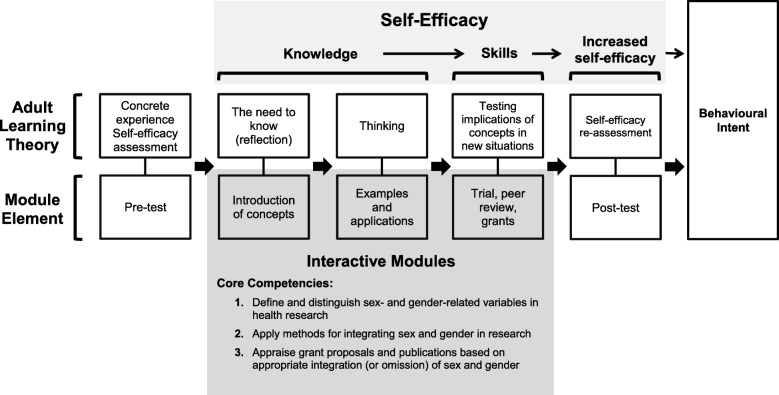
Conceptual model behind the development of the e-learning modules

Constructivist adult learning theories guided the sequence of each module with the aim of strengthening these core competencies (Fig. 1) [19]. The introduction and pre-test provide an opportunity for the learner to reflect on gaps in knowledge and skills. Part 1 invites the learner to fill these gaps through exposure to new concepts about sex and gender that are relevant to their discipline. Part 2 illustrates methods and teaches skills for operationalizing sex and gender appropriately in the conduct of the research and analysis. Part 3 tests the application of these skills using excerpts from real research protocols and publications. Learners are asked to write out the exact feedback and recommendations they would provide as a peer reviewer assessing the appropriate integration of sex and gender in a protocol or publication. Criteria are then listed so learners can self-assess whether their recommendations are sound with respect to the omission or inclusion of sex and gender, and how the study could be strengthened. The post-test reinforces knowledge gained and promotes self-efficacy for behavior change. Eighty percent of each module is interactive, with multiple-choice questions and pop-up answers. The modules were designed to be self-paced. Users can pause the modules at any time and return to complete them at a later time or date. After completing any of the three online modules and the post-test, the user receives an official personalized course completion certificate.

### Measurement of knowledge, self-efficacy, and behavior change intent

The pre- and post-test assessments each consist of six multiple-choice questions testing knowledge and skills, and three self-efficacy questions. For each module, the three competencies are evaluated with two knowledge questions and one self-efficacy question each. The knowledge questions differ based on the content of the module. The self-efficacy questions are identical, modeled on Bandura’s social-learning theory, and measure the confidence a person has in their ability to perform specific tasks or behaviors related to each of the three competencies (ability to distinguish sex- and gender-related variables, ability to apply sex and gender methods in research, and the ability to critically appraise the appropriate integration or omission of sex and gender in protocols and publications) [21]. Phrasing of the self-efficacy items and the response scaling was done in accordance with Bandura’s Conceptual Model of Self-Efficacy and his Guide for Constructing Self-Efficacy Scales [22]. All self-efficacy items begin with “How confident are you that you can …” followed by a description of the specific competency. Response options are presented as per Bandura’s guide on a 10-point horizontal visual analog scale with two anchors: 0 indicating “not at all confident I can do” and 10 indicating “extremely confident I can do.”

Both the pre- and post-test assessment questions are identical for each module. Only partial feedback is provided after completing the pre-test (i.e., the number of correctly answered knowledge questions). The correct responses to the knowledge questions are shared only after completing the post-test. As users are asked to self-rate self-efficacy for each competency area, three distinct pre- and corresponding post-test self-efficacy scores are available for each completed module.

Intent to change behavior was queried with a single item after completion of the post-test for each module. Participants were asked to endorse one of two response options to the statement “With respect to the approach I use in my own research program and publications for integrating sex and gender, I intend to: 1) Change the way I account for sex and gender; or 2) Not make any changes with respect to the way I integrate sex and gender.” Perceived added value of the training modules was also assessed with a single item after completion of the post-test. Participants were asked to endorse one of two response options to the statement “With respect to improving my knowledge and skills for integrating sex and/or gender in research this training module: 1) Taught me new knowledge and skills; or 2) Did not teach me anything new.”

Demographic data (age, occupation, country of origin) from each registered user were obtained prior to completion of the pre-test for each module.

### Data analysis

To assess knowledge gain, the number of correct responses on the knowledge pre- and post-test questions was calculated with 95% confidence intervals, for each competency, for each of the three modules. Comparisons between pre- and post-module test scores were performed using paired *t* tests. To assess gains in self-efficacy, self-rated scores were compared prior to and after completion of the module, with paired *t* tests. The distribution of participants who increased knowledge and self-efficacy, those who had a decrease in score and those that remained unchanged, were determined, along with 95% confidence intervals. Logistic regression was used to assess the odds of a change in behavioral intent due to increased knowledge or self-efficacy test scores, with adjustment for baseline scores. Linear regressions were used to assess whether participant demographics affected a change in score. All data were analyzed using R software version 3.4.0 (R Core Team (2017), https://www.R-project.org/).

## Results

### Participant demographics

Participant demographics for each training module are summarized in Table 1. A total of 543, 463, and 435 individuals completed the biomedical, primary data collection, and secondary data analysis e-learning modules, respectively. The greatest proportion of participants in each training module fell into the age category of 30–39 years (27.1%, 26.6%, and 29.7%). The majority were researchers (46.4%, 56.6%, and 57.2%), followed by researcher and peer reviewers, then trainees. Canada (81.0%, 88.1%, and 84.1%) was the most frequently reported country of origin, followed by the USA (11.4%, 4.8%, and 7.4%).

**Table 1:** Descriptive characteristics of training module participants

	Biomedical research *n* = 543 (%)	Data collection in humans *n* = 463 (%)	Analysis of human data *n* = 435 (%)
Age (years)			
≤ 29	107 (19.7%)	84 (18.1%)	77 (17.7%)
30–39	147 (27.1%)	123 (26.6%)	129 (29.7%)
40–49	125 (23.0%)	120 (25.9%)	120 (27.6%)
50–59	112 (20.6%)	92 (19.9%)	74 (17.0%)
≥ 60	51 (9.4%)	44 (9.5%)	35 (8.1%)
Occupation			
Researcher	252 (46.4%)	262 (56.6%)	249 (57.2%)
Researcher and peer reviewer	97 (17.9%)	84 (18.1%)	75 (17.2%)
Trainee	67 (12.3%)	45 (9.7%)	46 (10.6%)
Government employee	61 (11.2%)	31 (6.7%)	30 (6.9%)
Other	64 (11.8%)	41 (8.9%)	35 (8.1%)
Country			
Canada	440 (81.0%)	408 (88.1%)	366 (84.1%)
USA	62 (11.4%)	22 (4.8%)	32 (7.4%)
Asia or Europe	41 (7.6%)	33 (7.1%)	37 (8.5%)

### Change in knowledge and self-efficacy

Most participants displayed an increase in knowledge and self-efficacy (Table 2). For module 1, knowledge and self-efficacy post-test scores increased among 61.7% and 85.8% of participants, respectively. Module 2 knowledge post-test scores improved for 84.1% and self-efficacy scores improved for 76.5% of participants. For module 3, 73.1% and 81.6% of participants increased their knowledge and self-efficacy post-test scores, respectively. Knowledge scores displayed no change in 34.6%, 11.8%, and 21.3% of participants in modules 1, 2, and 3, respectively. There were no changes in self-efficacy scores in 11.7%, 15.9%, and 11.2% of participants in modules 1, 2, and 3.

**Table 2:** Distribution of changes in training module test scores

	Biomedical research % (95% confidence intervals)	Data collection in humans % (95% confidence intervals)	Analysis of human data % (95% confidence intervals)
Knowledge score			
Increase	61.7% (57.5%, 65.7%)	84.1% (80.4%, 87.1%)	73.1% (68.7%, 77.1%)
Decrease	3.7% (2.4%, 5.7%)	4.1% (2.7%, 6.4%)	5.6% (3.8%, 8.2%)
No change	34.6% (30.7%, 38.7%)	11.8% (9.1%, 15.1%)	21.3% (17.7%, 25.4%)
Self-efficacy score			
Increase	85.8% (82.5%, 88.6%)	76.5% (72.3%, 80.2%)	81.6% (77.5%, 85.1%)
Decrease	2.5% (1.5%, 4.3%)	7.6% (5.5%, 10.5%)	7.2% (5.1%, 10.2%)
No Change	11.7% (9.2%, 14.7%)	15.9% (12.8%, 19.6%)	11.2% (8.5%, 14.7%)

**Fig. 2:**
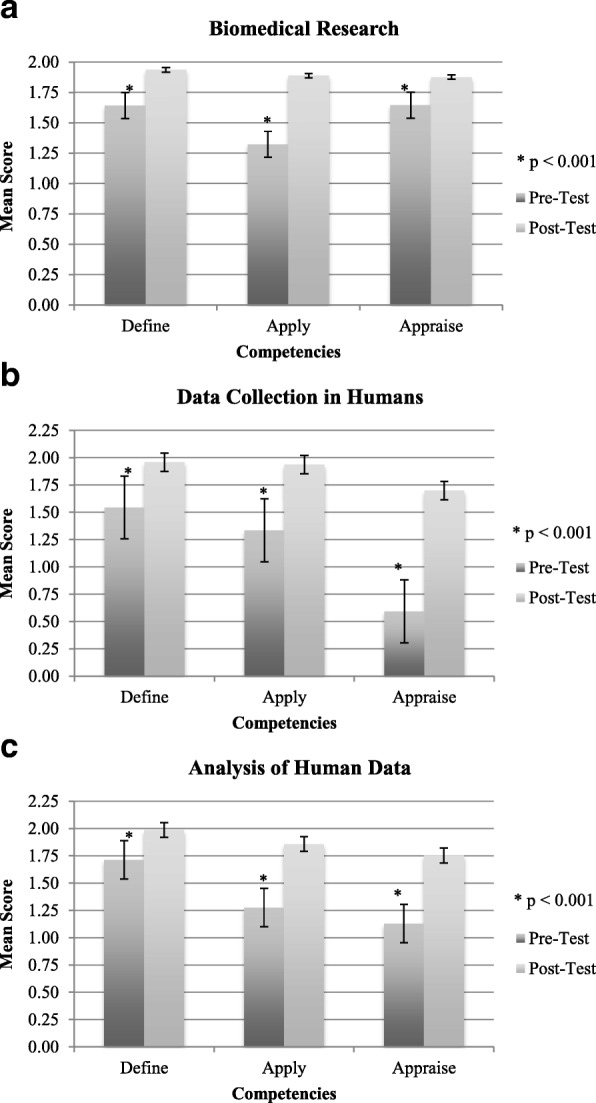
Knowledge improvement. **a**–**c** Pre- and post-test included the same six knowledge questions. Of these six questions, there were two questions per competency area, making the maximum score per competency 2.0. Error bars are the standard error of the mean. Significant differences (*p* < 0.001) from paired *t* tests are represented by asterisks (*)

**Fig. 3:**
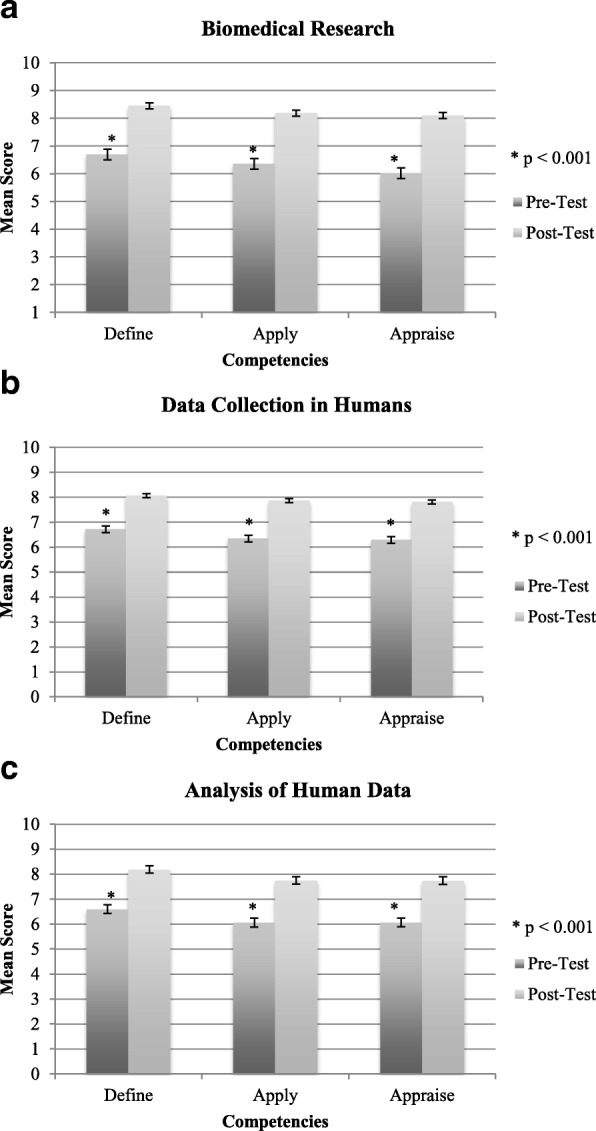
Self-efficacy improvement. **a**–**c** Pre- and post-test included the same three self-efficacy questions—one per competency area. For each question, participants rated their self-efficacy on a 10-point horizontal visual analog scale with two anchors: 0 indicating “not at all confident I can do” and10 indicating “extremely confident I can do” as per Bandura’s Guide for Constructing Self-Efficacy Scales [22]. Error bars are the standard error of the mean. Significant differences (*p* < 0.001) from paired *t* tests are represented by asterisks (*)

Comparison between pre- and post-learning knowledge and self-efficacy scores is presented in Figs. 2 and 3. Significant improvements in both knowledge and self-efficacy scores were noted for all three modules, across all competencies. The greatest gain in self-efficacy occurred among completers of the biomedical research module.

### Behavioral intent and perceived added value

The proportion of individuals indicating intent to change the way they account for sex and gender after completion of each e-learning module was 91.7%, 89.2%, and 94.0%, respectively. About 95.8%, 94.0%, and 96.3% of individuals perceived the training module they completed as having taught them new knowledge and skills. The odds of reporting an intent to change behavior increased by a factor of 1.29, 1.10, and 1.13 times for each 1-point increase in self-efficacy score for the biomedical, primary data collection, and secondary data analysis modules, respectively (Table 3). The association between an increase in knowledge and intent to change behavior was non-significant.

**Table 3:** Association between gains in knowledge and self-efficacy on self-reported intent to change the way sex and/or gender is accounted for in research

	Estimate^a^ OR, (95% CI)		
Variable	Module 1: Biomedical research	Module 2: Data collection in humans	Module 3: Analysis of human data
Increase in knowledge score^b^	1.38 (0.77, 2.31)	1.27 (0.80, 1.89)	1.20 (0.60, 2.15)
Increase in self-efficacy score^c^	1.29 (1.16, 1.43)	1.10 (1.00, 1.22)	1.13 (1.00, 1.28)

*OR* odds ratio, *CI* confidence intervals

^a^Adjusted for pre-test scores

^b^Continuous score out of 6, representing the six knowledge questions for each module

^c^Continuous score out of 30, representing the three self-efficacy questions for each module

### Effect of participant characteristics on improvement in test scores

Neither participant age, occupation, nor country of origin affected a change in post-test knowledge or self-efficacy scores, indicating that improvements were robust across learner profiles (data not shown).

## Discussion

E-learning was effective at increasing knowledge and self-efficacy for integrating sex and gender in health research and reporting. The majority of participants regardless of age or discipline acquired the three targeted competencies—(1) the ability to appropriately define and distinguish between sex-related versus gender-related variables, (2) the application of methods for integrating sex and gender, and (3) the critical appraisal of sex and gender integration in the design, methods, and analysis plan of research proposals and publications.

We believe there is a compelling rationale to mainstream the integration of sex and gender in health research and education. Not only are health research funding agencies, such as the National Institutes of Health and the Canadian Institutes of Health Research, calling for research proposals to account for sex and/or gender, but students expect it and patients deserve it [1, 2, 9, 23]. Sex-specific medication dosing is already recommended for drugs such as zolpidem and desmopressin [24, 25], and many physiologic parameters differ by sex [9]. As the body of evidence grows in favor of sex- and gender-specific pathophysiologic mechanisms, the management of patients for a variety of health conditions will need to keep pace with the changing nature of science [26].

Several resources are available to help educators embed concepts of sex and gender into graduate and postgraduate curricula [27, 28]. However, to our knowledge, the Institute of Gender and Health e-learning modules are the first to undergo rigorous testing for evaluating changes in knowledge and self-efficacy. E-learning is flexible, with content that is dynamic, changing, self-paced, and that ultimately enhances the sustainability of knowledge transfer [29]. The approach is ideal for standardizing sex and gender integration across settings and countries. Furthermore, the modules are free and widely accessible. To date, over 3000 academics, students, and public servants have completed the e-learning modules. Although e-learning cannot replace in-person mentoring and coaching, educators and supervisors can use the modules to support curriculum development efforts across health research disciplines.

A strength of this study was objective assessment of changes in knowledge and self-efficacy as a result of completing the e-learning modules. Gains in knowledge were statistically significant and provided clear evidence of participants having acquired competencies around defining and distinguishing sex and gender, applying methods to account for sex and gender in research, and appraising research protocols and publications. Changes in self-efficacy are harder to interpret, as they represent an individual’s judgment about their level of competence or ability to successfully perform a behavior [21]. As a primary construct of social cognitive theory, self-efficacy is one of the constructs most frequently used to predict, explain, and change behaviors [30]. Although self-efficacy scores improved in a statistically significant fashion in this evaluation, they rarely reached a score of 10 (full confidence). Interestingly, improvements in self-efficacy, but not knowledge, were associated with self-reported behavioral intent to change the way sex and gender was accounted for in research.

There are limitations to the current analysis. First, although the format of self-efficacy assessments is well accepted and valid, the knowledge questions in the pre- and post-tests were not previously tested and may not capture adequately the sex and gender competency constructs under study. Second, we queried self-reported behavioral intent as a single item question at the end of the post-test for each module. Behavioral intent is a subjective measure and is likely correlated with applicants’ motivation to complete the modules and possible propensity for e-learning. We do not know if completion of the modules translated into actual, objective changes in practice or future impact in a given field. The methods for recruiting participants may have led to enrolment of a biased sample of researchers already interested in the field of sex and gender in science, which may explain the relatively high pre-test scores for some of the knowledge items as well as the high rate of endorsement of the statement about changing behavior. Finally, assessments occurred once, immediately after completion of the modules. We did not assess whether the observed gains in knowledge and self-efficacy were sustainable over time. However, as most students’ satisfaction questionnaires are based on self-report, we believe that the methods used to assess the e-learning modules compare favorably with other course evaluations, with over 94% of completers endorsing the modules as being helpful for increasing their skills and understanding.

## Conclusion

Funders, academic institutions, and professional societies are uniquely positioned to leverage continuing education to enhance the quality of health research and practice. As such, the Institute of Gender and Health’s e-learning modules have become a mandatory eligibility requirement for applicants submitting research proposals to an increasing number of priority-driven funding competitions in Canada. Promoting evidence-based capacity building methods that have been shown to increase knowledge and self-efficacy for integrating sex and gender in academic activities is an active step towards enhancing rigor, reproducibility, and inclusion. It is our hope that the e-learning modules will contribute to improving the uptake of sex and gender considerations in health research, policy, and practice.
